# Supplementary material for: Geometry can provide long-range mechanical guidance for embryogenesis
Source: PLoS Comput Biol. 2017 Mar 27;13(3):e1005443. doi: 10.1371/journal.pcbi.1005443 (PMC5386319; doi:10.1371/journal.pcbi.1005443)
Supplement: S1 Text — (PDF) [file pcbi.1005443.s001.pdf]

Supplementary Text of the papaer
Geometry can provide long-range mechanical guidance for embryogenesis

Mahamar Dicko[1–4] Pierre Saramito[1,3] Guy Blanchard[5] Claire Lye[5]

Bénédicte Sanson[5] Jocelyn Étienne[2,4]

[1]LJK and [2]LIPHY, Univ. Grenoble Alpes, France
[3]LJK and [4]LIPHY, CNRS, France
[5]PDN, University of Cambridge, United Kingdom

Lagrange multiplier approach for tangential flows

We present a generic finite element approach allowing to address flows of finite surface-compressibility η_{b}. We approximate the solution of vectorial equations set on a curved surface of $\mathbb{R}^3$. This also offers a framework for future extensions of the method to non-Newtonian fluids.

Eq (3) must be solved for velocities $\boldsymbol{v}$ tangential to the surface Γ, which corresponds to the continuum formed by the apical actomyosin cortices of cells and adherens junctions. In terms of solution spaces, this constraint can be written as $\boldsymbol{v} \in \boldsymbol{V}_t = \left\{ \boldsymbol{w} \in \left(H^1(\Gamma)\right)^3 \mid \boldsymbol{w} \cdot \boldsymbol{n} = 0 \right\}$, where $\left(H^1(\Gamma)\right)^3$ is the set of vector-valued functions defined on Γ whose differential is square-integrable, and $\boldsymbol{n}$ is the outer normal to Γ. Constraints such as the tangentiality of the solution can be implemented either by defining finite element spaces that can satisfy the constraint (see e.g. the work by [1]) or by introducing a mixed finite element approximation following e.g. [2]. Here we opt for the latter solution, which allows us to discretise vector fields on the surface in the Cartesian system of coordinates rather than a curvilinear one.

Using an energetic formulation, it can then be shown that Eq (3) is equivalent to the constrained minimisation:

$$\boldsymbol{v} = \arg \inf_{\boldsymbol{w} \in \boldsymbol{V}_t} E(\boldsymbol{w}) \tag{1}$$

where E is the rate of energy dissipation in the tissue, namely:

$$E(\boldsymbol{w}) = \int_\Gamma \frac{c_{\mathrm{f}}}{2} |\boldsymbol{w}|^2 \,\mathrm{ds} + \int_\Gamma \eta \,|\dot{\varepsilon}(\boldsymbol{w})|^2 \,\mathrm{ds} + \int_\Gamma \frac{\eta_{\mathrm{b}}}{2} |\nabla_\Gamma \cdot \boldsymbol{w}|^2 \,\mathrm{ds} - \int_\Gamma \boldsymbol{f} \cdot \boldsymbol{w} \,\mathrm{ds}$$

and $\boldsymbol{f} = \boldsymbol{\nabla}_\Gamma \cdot \boldsymbol{\sigma}_{\mathbf{a}}$.

Our approach is to introduce a vector field $\boldsymbol{\theta}$ that will act as a Lagrange multiplier to constrain the velocities $\boldsymbol{v}$ to be tangential. This field $\boldsymbol{\theta}$ can be interpreted as the force needed to prevent normal deformations. In order to do this, we first define $\boldsymbol{\theta}$ by $\boldsymbol{\theta} = \gamma \boldsymbol{L}(\boldsymbol{v})$ where $\boldsymbol{L}(\boldsymbol{v}) = (\boldsymbol{v} \cdot \boldsymbol{n})\,\boldsymbol{y} - \nabla_\Gamma \boldsymbol{v} \cdot \boldsymbol{n}$, $\boldsymbol{y}$ is the curvature vector defined as $\boldsymbol{y} = \mathrm{Tr}(\nabla_\Gamma \boldsymbol{v})\boldsymbol{n}$, and γ is a strictly positive parameter. Then we note that $\boldsymbol{V}_t = \ker \boldsymbol{L}$. The problem can now be rewritten as an unconstrained saddle-point problem:

$$(\boldsymbol{v}, \boldsymbol{\theta}) = \arg \inf_{\boldsymbol{w} \in \boldsymbol{V}} \sup_{\boldsymbol{\xi} \in \boldsymbol{\Xi}} E(\boldsymbol{w}) + \int_\Gamma \left(\boldsymbol{L}(\boldsymbol{w}) - \frac{1}{\gamma} \boldsymbol{\xi} \right) \cdot \boldsymbol{\xi} \,\mathrm{ds}$$

where $\boldsymbol{V} = \left(H^1(\Gamma)\right)^3$ and $\boldsymbol{\Xi} = \left(L^2(\Gamma)\right)^3$, the set of square-integrable vector fields. We further introduce the surface pressure p which enforces the finite compressibility of the actomyosin in the tangential plane,

$p = -\eta_{\mathrm{b}} \nabla_\Gamma \cdot \boldsymbol{v}$. We can then write the problem as :

$$(\boldsymbol{v}, p, \boldsymbol{\theta}) = \arg \inf_{\boldsymbol{w} \in \boldsymbol{V}} \sup_{\substack{q \in Q \\ \boldsymbol{\xi} \in \boldsymbol{\Xi}}} \mathcal{L}(\boldsymbol{w}, q, \boldsymbol{\xi})$$

where $Q = L^2(\Gamma)$,

$$\mathcal{L}(\boldsymbol{w}, q, \boldsymbol{\xi}) = \frac{1}{2} a(\boldsymbol{w}, \boldsymbol{w}) + b_1(\boldsymbol{w}, q) - \frac{1}{2} c_1(q, q) + b_2(\boldsymbol{w}, \boldsymbol{\xi}) - \frac{1}{2} c_2(\boldsymbol{\xi}, \boldsymbol{\xi}) - \ell(\boldsymbol{w})$$

and a, b_1, b_2, c_1 and c_2 are the bilinear forms defined by :

$$\begin{aligned} a(\boldsymbol{v}, \boldsymbol{w}) &= \int_\Gamma c_{\mathrm{f}} \boldsymbol{v} \cdot \boldsymbol{w} \, \mathrm{ds} + \int_\Gamma 2\eta \dot{\boldsymbol{\varepsilon}}(\boldsymbol{v}) : \dot{\boldsymbol{\varepsilon}}(\boldsymbol{w}) \, \mathrm{ds}, \\ b_1(\boldsymbol{w}, q) &= \int_\Gamma -q \nabla_\Gamma \cdot \boldsymbol{w} \, \mathrm{ds}, \quad c_1(p, q) = \int_\Gamma \frac{1}{\eta_{\mathrm{b}}} pq \, \mathrm{ds}, \\ b_2(\boldsymbol{w}, \boldsymbol{\xi}) &= \int_\Gamma \boldsymbol{L}(\boldsymbol{w}) \cdot \boldsymbol{\xi} \, \mathrm{ds}, \quad c_2(\boldsymbol{\theta}, \boldsymbol{\xi}) = \int_\Gamma \frac{1}{\gamma} \boldsymbol{\theta} \cdot \boldsymbol{\xi} \, \mathrm{ds} \end{aligned}$$

and ℓ is the linear form define by : $\ell(\boldsymbol{w}) = \int_\Gamma \boldsymbol{f} \cdot \boldsymbol{w} \, \mathrm{ds}$. The saddle point can then be characterized as the solution of the linear problem :

$$\begin{array}{lllllll} a(\boldsymbol{v}, \boldsymbol{w}) & + & b_1(\boldsymbol{w}, p) & + & b_2(\boldsymbol{w}, \boldsymbol{\theta}) & = & \ell(\boldsymbol{w}) & \forall\, \boldsymbol{w} \in \left(H^1(\Gamma)\right)^3 \\ b_1(\boldsymbol{v}, q) & - & c_1(p, q) & & & = & 0 & \forall\, q \in Q \\ b_2(\boldsymbol{v}, \boldsymbol{\xi}) & & & - & c_2(\boldsymbol{\theta}, \boldsymbol{\xi}) & = & 0 & \forall\, \boldsymbol{\xi} \in \Xi. \end{array}$$

We show [3] that this problem has a unique solution in $\boldsymbol{V} \times \boldsymbol{\Xi} \times Q$.

Mixed finite element approach

We solve the saddle point problem using the finite element method. This requires us to introduce a mesh Γ_h approximating Γ. We use a triangular tessellation of second order, i.e. elements are curved triangles described by a quadratic transformation and whose largest dimension is smaller than the mesh size h. This ensures that the distance between any point of Γ_h and Γ is at most Ch^3, where C is a constant independent of h. Using this mesh, we define discrete functional spaces $\boldsymbol{V}_h$, $\boldsymbol{\Xi}_h$ for vector fields $\boldsymbol{v}_h$, $\boldsymbol{\theta}_h$ and Q_h of scalar field p_h. We approach the saddle point problem using the following formulation :

$$\begin{array}{lllllll} a_h(\boldsymbol{v}_h, \boldsymbol{w}_h) & + & b_{1,h}(\boldsymbol{w}_h, p_h) & + & b_{2,h}(\boldsymbol{w}_h, \boldsymbol{\theta}_h) & = & \ell_h(\boldsymbol{w}_h) & \forall\, \boldsymbol{w}_h \in \boldsymbol{V}_h \\ b_{1,h}(\boldsymbol{v}_h, q_h) & - & c_{1,h}(p_h, q_h) & & & = & 0 & \forall\, q_h \in Q_h \\ b_{2,h}(\boldsymbol{v}_h, \boldsymbol{\xi}_h) & & & - & c_{2,h}(\boldsymbol{\theta}_h, \boldsymbol{\xi}_h) & = & 0 & \forall\, \boldsymbol{\xi}_h \in \boldsymbol{\Xi}_h \end{array}$$

where $a_h, b_{1,h}, b_{2,h}, c_{1,h}$ and $c_{2,h}$ are bilinear forms approximating the original forms, defined by :

$$\begin{aligned} a_h(\boldsymbol{v}_h, \boldsymbol{w}_h) &= \int_{\Gamma_h} c_{\mathrm{f}} \boldsymbol{v}_h \cdot \boldsymbol{w}_h \, \mathrm{ds}_h + \int_{\Gamma_h} 2\eta \dot{\boldsymbol{\varepsilon}}_h(\boldsymbol{v}_h) : \dot{\boldsymbol{\varepsilon}}_h(\boldsymbol{w}_h) \, \mathrm{ds}_h, \\ b_{1,h}(\boldsymbol{w}_h, q_h) &= \int_{\Gamma_h} -q_h \nabla_{\Gamma_h} \cdot \boldsymbol{w}_h \, \mathrm{ds}_h, \quad c_{1,h}(p_h, q_h) = \int_{\Gamma_h} \frac{1}{\eta_{\mathrm{b}}} p_h q_h \, \mathrm{ds}_h, \\ b_{2,h}(\boldsymbol{w}_h, \boldsymbol{\xi}_h) &= \int_{\Gamma_h} \boldsymbol{L}_h(\boldsymbol{w}_h) \cdot \boldsymbol{\xi}_h \, \mathrm{ds}_h, \quad c_{2,h}(\boldsymbol{\theta}_h, \boldsymbol{\xi}_h) = \int_{\Gamma_h} \frac{1}{\gamma} \boldsymbol{\theta}_h \cdot \boldsymbol{\xi}_h \, \mathrm{ds}_h \end{aligned}$$

and ℓ_h is the linear form defined by $\ell_h(\boldsymbol{v}_h) = \int_{\Gamma_h} \boldsymbol{f}_h \cdot \boldsymbol{v}_h \, \mathrm{ds}_h$. The choice of the finite element spaces cannot be made arbitrarily because it is a mixed problem. It requires a suitable choice in order for the discrete problem converges towards the saddle point problem. Indeed, the discrete problem must verify

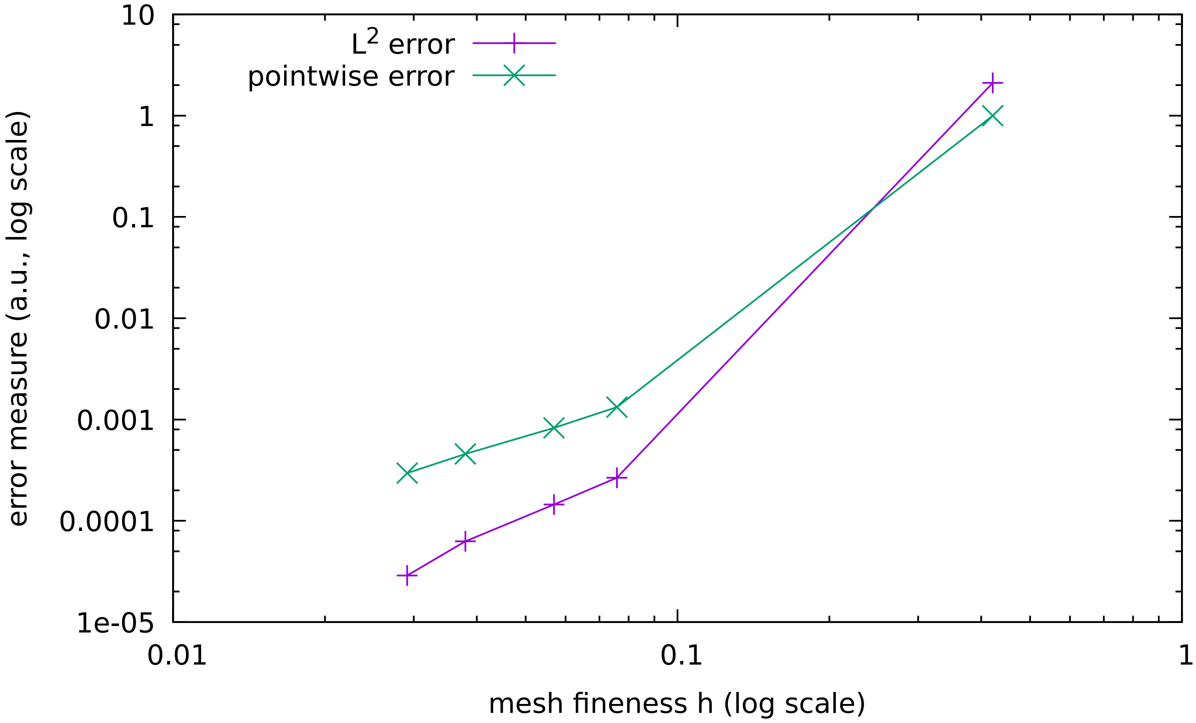

Figure 1: Convergence test. The mesh is refined (from right to left) and the error on an arbitrary flow field is seen to decrease. L^2 error is the overall squared difference of calculated minus original velocity vectors, pointwise (L^∞) error is the length of the largest difference between calculated and original velocity vectors over the whole mesh.

---

two conditions called *inf–sup* or *Brezzi–Babuska* conditions (see [4]) : first between the spaces $\boldsymbol{V}_h$ and Q_h through the bilinear form $b_{1,h}$, then between the spaces $\boldsymbol{V}_h$ and $\boldsymbol{\Xi}_h$ through the bilinear form $b_{2,h}$. In the absence of theoretical results on spaces that may verify these conditions, the idea is to produce compatible mixed finite element combinations in order to obtain the convergence. For this, we guided our choice by similarity with choices for which *inf–sup* conditions are verified in the case of classical problems (such as the three-dimensional Stokes problem).

Numerical validation

Next, finite element spaces $\boldsymbol{V}_h$, Q_h and $\boldsymbol{\Xi}_h$ must be specified. We base them on a triangular tesselation of the surface Γ (see next section) and choose Lagrange finite elements of degree 3 for $\boldsymbol{V}_h$, 2 for Q_h and $\boldsymbol{\Xi}_h$. We then check that this choice leads to a convergent approximation of the solution of Eq (3). In order to do so, we make an arbitrary choice of a velocity field on an arbitrary surface (a sphere), and calculate analytically the prestress that would be needed to achieve such a velocity field. We then run simulations on a series of meshes of decreasing triangle size h and monitor the evolution of the error $\boldsymbol{v} - \boldsymbol{v}_h$. We show [3] that this decreases quadratically when h decreases, leading to pointwise errors (i.e., in L^∞ norm) smaller than 10^{-3} for all meshes of more than 10^4 elements ($h = 0.05$). For these numerical tests, we chose : $c_\mathrm{f} = 10^{-5}$, $\eta = 1$, $\eta_\mathrm{b} = 10^3$ and $\gamma = 10^7$.

Finite element mesh of the *Drosophila* embryo and resolution

We first describe the embryo shape with an analytical function, and then introduce a procedure to create a finite element mesh which will be fine enough to capture geometric details such as the cephalic furrow,

while remaining of reasonable size in terms of the number of triangles (since the computational cost of our algorithms increases like $N\log(N)$ with number of triangles N).

The analytical function describing the embryo shape $\Gamma = \{\phi(x,y,z)=0\}$ is chosen as:

$$\phi(x,y,z) = 1 - \sqrt{\left(\frac{x}{R_{AP}}\right)^2 + \left(\frac{y}{R_{DV}}\right)^2 + \left(\frac{z-\frac{1}{2}c_{AP}x^2}{R_{DV}}\right)^2} + D_{CF}\psi_{CF}\left(\frac{x - S_{CF}z - x_{CF}}{W_{CF}}\right)$$

where R_{AP} is the half-length of the embryo in AP, R_{DV} its maximum radius in a transverse cut, c_{AP} a curvature parameter corresponding to the curvature of the main axis of the embryo (defined as the locus of the centre of all transverse cuts), and parameters indexed with CF correspond to the cephalic furrow. When $D_{CF} = 0$, the cephalic furrow is absent, and the geometry corresponds to an ellipsoid of major axis along x, with radius R_{AP}, and minor axes along y and z of equal radii R_{DV}. The curvature parameter flattens the dorsal side ($z > 0$). We take $R_{DV} = 1$ as the reference adimensional length, $R_{AP} = 3R_{DV}$ and $c_{AP} = 0.1/R_{DV}$, which leads to a shape close to the one of actual embryos.

The cephalic furrow depth is described by $D_{CF} = 0.1R_{DV}$, its position along the x axis in the mid-coronal plane $z = 0$ is given by $x_{CF} = -1.2R_{DV}$, and its inclination with respect to the (y,z) transverse planes is set by $S_{CF} = 0.3$. The cephalic furrow has a total width $W_{CF} = 0.1R_{DV}$ (exaggerated compared to real embryos, since a very thin and sharp feature would increase tremendously the computational cost), its shape is described by the function

$$\psi_{CF}(s) = \begin{cases} \exp\left(-\frac{2}{1-s^2}+2\right) & \text{if } |s|<1 \\ 0 & \text{else} \end{cases}$$

which is infinitely derivable, leading to a very smooth profile.

The mesh generation is delegated to `mmgs` software [5], and the meshes used have around 46000 elements. The numerical resolution of the problem on this mesh is implemented in the open-source free software environment `rheolef` [6].

Microscopy and cell tracking

In Fig 1*a* we present data extracted from the tracking of GB extension in a wildtype embryo with the whole-membrane markers *resille-GFP* and *spider-GFP*, as described in [7]. Imaging was done using multidirectional selective plane illumination microscopy (mSPIM) [8], embryos were rotated to image four perpendicular views which were reconstructed into a whole embryo image stack post acquisition [9]. Image stacks were acquired every 30 seconds.

In order to monitor the displacement of each cell, we first sensed the shape of the surface of the embryo in the three-dimensional mSPIM z-stack. We applied a grey-scale threshold to binarise the z-stack, highlighting only cell membrane-labelled signal. A 'blanket' of a fine meshwork of line-segments was dropped in the positive z direction down onto the embryo, until caught by cell membrane signal. This described the surface of the embryo accurately with a curved mesh, located at the apices of the embryonic epithelium. We used this embryo surface 'blanket' to extract curved image layers, with deeper layers shrinking progressively in single pixel steps in the direction normal to the local embryonic surface, towards the centre of the embryo [10]. The radial depth giving the clearest view of the cell outlines of the embryonic epithelium was selected for tracking. Automated cell tracking with manual correction was performed using custom software written in 'C' and Interactive Data Language (IDL, Harris Geospatial). This tracked all cells in the chosen curved layer over time, identifying cell outline shapes and links forwards and backwards in time in an iterative process using an adaptive watershedding algorithm [11, 12]. From each cell shape we calculated the location of the cell centroid (centre of mass). The coordinates of cell centroids, perimeter shapes, and links forwards and backwards in time were stored for all cells at each time point.

Using the relative movements of cell centroids, local tissue 2D rate-of-strain tensor (deformation rate) was calculated for small spatio-temporal domains focused on every tracked cell at each time point [11]. Local domains were composed of a focal cell and one corona of neighbouring cells over a 30 s interval (between successive frames). Local domains were first un-tilted according to the orientation of the local embryo

surface and stretched to minimize artifacts caused by the local Gaussian curvature. All strain rates were then projected onto the embryonic axes, AP and DV. The rate of local area change was calculated as the trace of the 2D tissue strain rate tensor.

References

1. Destuynder P, Salaun M. A mixed finite element for shell model with free edge boundary condition. Part 1. The mixed variational formulation. Comput Methods Appl Mech Engrg. 1995;120:195–217.

2. Brezzi F, Fortin M. Mixed and hybrid finite elements methods. New-York: Springer-Verlag; 1991.

3. Dicko M. Méthodes numériques pour la résolution d'EDP sur des surfaces. Application dans l'embryogenèse. Univ. Grenoble; 2016.

4. Brezzi F, Fortin M. Mixed and hybrid finite element methods. New York, NY, USA: Springer-Verlag New York, Inc.; 1991.

5. Dapogny C, Dobrzynski C, Frey P. Three-dimensional adaptive domain remeshing, implicit domain meshing, and applications to free and moving boundary problems. J Comput Phys. 2014;262:358–378.

6. Saramito P, Roquet N, Étienne J. `rheolef`, a C++ finite element environment. CNRS; 2003–2014.

7. Lye CM, Blanchard GB, Naylor H, Muresan L, Huisken J, Adams R, et al. Mechanical coupling between endoderm invagination and axis extension in Drosophila. PLoS Biol. 2015;13:e1002292.

8. Huisken J, Stainier DY. Selective plane illumination microscopy techniques in developmental biology. Devel. 2009;136:1963–1975.

9. Preibisch S, Saalfeld S, Schindelin J, Tomancak P. Software for a bead-based registration of selective plane illumination microscopy data. Nature Methods. 2010;7:418–419.

10. England SJ, Blanchard GB, Mahadevan L, Adams RJ. A dynamic fate map of the forebrain shows how vertebrate eyes form and explains two causes of cyclopia. Devel. 2006;133:4613–4617.

11. Blanchard GB, Kabla AJ, Schultz NL, Butler LC, Sanson B, Gorfinkiel N, et al. Tissue tectonics: morphogenetic strain rates, cell shape change and intercalation. Nature Methods. 2009;6:458–464.

12. Butler LC, Blanchard GB, Kabla AJ, Lawrence NJ, Welchman DP, Mahadevan L, et al. Cell shape changes indicate a role for extrinsic tensile forces in Drosophila germ-band extension. Nature Cell Biol. 2009;11:859–864.
